# Lower Trabecular Volumetric BMD at Metaphyseal Regions of Weight-Bearing Bones is Associated With Prior Fracture in Young Girls

**DOI:** 10.1002/jbmr.218

**Published:** 2010-08-18

**Authors:** Joshua N Farr, Rita Tomás, Zhao Chen, Jeffrey R Lisse, Timothy G Lohman, Scott B Going

**Affiliations:** 1 Department of Physiological Sciences, University of Arizona Tucson, AZ, USA; 2 Department of Nutritional Sciences, University of Arizona Tucson, AZ, USA; 3 Department of Physical Medicine and Rehabilitation, Hospital Curry Cabral Lisbon, Portugal; 4 Department of Epidemiology and Biostatistics, University of Arizona Tucson, AZ, USA; 5 Department of Medicine, University of Arizona Tucson, AZ, USA

**Keywords:** FRACTURE, YOUTH, BONE GEOMETRY, VOLUMETRIC BONE MINERAL DENSITY (VBMD), PERIPHERAL QUANTITATIVE COMPUTED TOMOGRAPHY (PQCT)

## Abstract

Understanding the etiology of skeletal fragility during growth is critical for the development of treatments and prevention strategies aimed at reducing the burden of childhood fractures. Thus we evaluated the relationship between prior fracture and bone parameters in young girls. Data from 465 girls aged 8 to 13 years from the Jump-In: Building Better Bones study were analyzed. Bone parameters were assessed at metaphyseal and diaphyseal sites of the nondominant femur and tibia using peripheral quantitative computed tomography (pQCT). Dual-energy X-ray absorptiometry (DXA) was used to assess femur, tibia, lumbar spine, and total body less head bone mineral content. Binary logistic regression was used to evaluate the relationship between prior fracture and bone parameters, controlling for maturity, body mass, leg length, ethnicity, and physical activity. Associations between prior fracture and all DXA and pQCT bone parameters at diaphyseal sites were nonsignificant. In contrast, lower trabecular volumetric BMD (vBMD) at distal metaphyseal sites of the femur and tibia was significantly associated with prior fracture. After adjustment for covariates, every SD decrease in trabecular vBMD at metaphyseal sites of the distal femur and tibia was associated with 1.4 (1.1–1.9) and 1.3 (1.0–1.7) times higher fracture prevalence, respectively. Prior fracture was not associated with metaphyseal bone size (ie, periosteal circumference). In conclusion, fractures in girls are associated with lower trabecular vBMD, but not bone size, at metaphyseal sites of the femur and tibia. Lower trabecular vBMD at metaphyseal sites of long bones may be an early marker of skeletal fragility in girls. © 2011 American Society for Bone and Mineral Research.

## Introduction

Fractures are common during growth, occurring in approximately one in three children who are otherwise healthy.(1–4) Moreover, children who sustain a fracture are two- to threefold more likely to fracture again compared with those who are fracture-free.(5) In children and adolescents, fractures are the leading cause of hospital admission following injury.(6) Fracture incidence in youth has accelerated over the past 30 years,(7) and it has become increasingly important to understand the etiology of bone fragility so that prevention strategies can be developed.

Fractures during growth may be associated with skeletal fragility. For example, bone mineral content (BMC, g) and areal bone mineral density (aBMD, g/cm^2^) have been shown to be lower in children with prior fracture than in age-matched controls.(8–12) Furthermore, prospective studies have shown that childhood fractures are associated with less gain of BMC and volumetric BMD (vBMD, mg/cm^3^) during puberty.(13–16) Since BMD and bone geometry tend to track throughout life in the percentile of origin during growth,(17) lower BMD and unfavorable bone geometry may be predictors of fracture during growth and later in life.

Although fractures can occur at any time during growth, peak incidence coincides with age of peak height velocity (PHV).(4,18) Metaphyseal regions of long bones may be particularly prone to fracture during puberty.(1–4) Some experts have hypothesized that enhanced bone turnover during peak longitudinal growth leads to increased intracortical porosity, transiently causing a lag in bone strength and leaving the already thin metaphyseal cortex susceptible to fracture.(19) Others have hypothesized that endocortical apposition lags during peak linear growth, leading to decreased cortical thickness and increased susceptibility to fracture.(20,21) The forearm has received much attention because it is the most common site of fracture in children.(1–4) For example, Cheng and colleagues(16) studied pubertal girls and reported that lower vBMD but not cross-sectional area (CSA) of the distal radius was associated with prior forearm fracture and that the lower vBMD deficit persisted 7 years later. However, whether vBMD, geometry, or both are compromised in children at metaphyseal regions of other long bones remains unclear. In young-adult males (aged ∼19 years) with prior fracture, Darelid and colleagues(18) recently reported lower trabecular vBMD at metaphyseal sites but no difference in bone geometry at diaphyseal sites of the radius and tibia. Thus we sought to ascertain whether this relationship is also evident at metaphyseal and diaphyseal sites of the distal femur and tibia in young girls who had suffered at least one fracture compared with girls who were fracture-free.

## Methods

### Participants

Baseline data were analyzed for 465 healthy girls, aged 8 to 13 years, who were participants in the Jump-In: Building Better Bones study.(22) The long-term goal of the Jump-In study is to prospectively assess the effects of high-impact jumping exercises on bone macroarchitecture in pre- and early-pubertal girls. Girls who were in school grade 4 or 6 were recruited from 14 elementary and 4 middle schools around Tucson, Arizona. Exclusion criteria included learning disabilities (identified by schools) that made it impossible to complete questionnaires or otherwise unable to comply with assessment protocols; medications, medical conditions, or a disability that limited participation in physical exercise as defined by the Committee on Sports Medicine and Fitness(23); excluded (or excused) from participation in physical education; and the inability to read and understand English. The protocol was approved by the University of Arizona Human Subjects Protection Committee, and the study was conducted in accordance with the Helsinki Declaration. All parents/guardians and girls provided written informed consent. Parents/guardians completed a questionnaire that inquired about participant ethnicity and race. A standardized questionnaire was used to collect information about smoking habits, and the Past Year Physical Activity Questionnaire (PYPAQ)(24,25) was used to collect information about the average duration and frequency of physical activity participation. The PYPAQ has been validated in adolescents.(26) We used a modified version of the PYPAQ that has been described in detail previously.(24,25) Total PYPAQ score was computed using an equation from Shedd and colleagues(27): PYPAQ score = ∑_1–*n*_[duration (minutes/session) × frequency (days/week) × load (peak strain score(28))], where *n* is the number of activities a subject reported during the past year.

### Fracture history (self-reported)

A parent/guardian of each girl completed a health history questionnaire that inquired about all injuries suffered by their child that required medical attention. The questionnaire also inquired about any injuries that resulted in fracture and the number of fractures suffered. Then, for each individual fracture, detailed information was obtained on the bone(s) afflicted, the date of the incident, and whether surgery was needed.

### Anthropometry

Anthropometric measures were obtained following standardized protocols(29). Body mass was measured to the nearest 0.1 kg using an electronic scale (Seca, Model 881, Hamburg, Germany), and height and sitting height were measured at full inhalation to the nearest millimeter using a stadiometer (Shorr Height Measuring Board, Olney, MD, USA). Nondominant femur length (nearest millimeter) was measured from the base of the patella to the inguinal crease. Nondominant tibia length (nearest millimeter) was measured from the proximal end of the medial border of the tibial plateau to the distal edge of the medial malleolus. Coefficients of variation (CVs) for femur and tibia lengths were 0.33% and 0.51%, respectively (*n* = 465).

### Physical maturation

Maturity was assessed from self-report (with assistance available) of breast development based on Tanner stages. The questionnaire presents illustrations of stages of development and has been validated(30) and shown to agree with physician exam and grading. Although Tanner staging is common in developmental studies, its ability to accurately assess maturation has been shown to be limited.(31) Consequently, we also assessed an index of maturation, estimated years from peak height velocity (PHV), using Mirwald's equation(32) derived from data from a 6-year longitudinal study in boys and girls.(33) In Mirwald's sample, the equation for girls explained 89% of the variance in years from PHV.(32)

### Bone and body composition assessment

Bone geometry and vBMD were assessed at the distal 4% and 20% femur and 4% and 66% tibia sites of the nondominant leg using peripheral quantitative computed tomography (pQCT; XCT 3000; Stratec Medizintechnik GmbH, Pforzheim, Germany, Division of Orthometrix; White Plains, NY, USA). Subjects were asked with which foot they would kick a ball when playing soccer/kickball. If the subject was uncertain, she was asked with what hand she writes, and that was determined to be her side of dominance. Scout scans were performed to locate the distal growth plates, with the scanner programmed to subsequently find the sites of interest. pQCT scans were analyzed using Stratec software, Version 6.0. At the distal metaphyseal sites of the femur and tibia, we used Contour mode 3 (169 mg/cm^3^) to define the total bone, and Peel mode 4 (650 mg/cm^3^ with a 10% peel) was used to ensure that only trabecular bone remained. Because of the difficulties in interpreting metaphyseal bone density measurements from a single slice,(34) we averaged three pQCT slices at both the femur and tibia 4% sites. At the diaphyseal 20% femur and 66% tibia sites, Contour mode 1 (710 mg/cm^3^), Peel mode 2 (710 mg/cm^3^), and Cort mode 2 (710 mg/cm^3^) were used. Further details on image processing, calculations, and analysis, including descriptions of Contour, Peel, and Cort modes, are published elsewhere.(35) Slice thicknesses were 2.3 mm, and voxel size was set at 0.4 mm for all sites. Scanner speed was set at 25 mm/second. Trabecular vBMD (mg/cm^3^) and periosteal circumference (PC, mm) were assessed at the 4% femur and tibia sites, whereas cortical vBMD (mg/cm^3^), endosteal circumference (EC, mm), PC, and cortical thickness (mm) were assessed at the 20% femur and 66% tibia.

Areal BMD (g/cm^2^), BMC (g), and bone area (BA, cm^2^) of the total body less head (TBLH), L_2_–L_4_ lumbar spine (LS) vertebrae in the anteroposterior plane, femoral neck (FN), and total femur and tibia of the nondominant leg were assessed with dual-energy X-ray absorptiometry (DXA) using a GE Lunar Prodigy (software Version 5.60.003) fan-beam densitometer (GE Lunar Corp, Madison, WI, USA). Subjects were positioned using standard GE/Lunar protocols. Total-body mass, fat mass, percent total-body fat, and lean soft tissue mass were obtained from DXA whole-body scans. Because of the inherent limitations of aBMD in children and adolescents,(36) and because the head is not responsive to environmental stimuli such as physical activity,(1) TBLH BMC has been proposed by the International Society for Clinical Densitometry (ISCD) as the most appropriate DXA-derived outcome measure of bone status in youth.(37)

Calibration and quality assurance of the pQCT and DXA instruments were performed daily to ensure the accuracy and precision of measurements. Operators were trained on pQCT scanning and software analyses following guidelines provided by Bone Diagnostics, Inc. (Fort Atkinson, WI, USA). One operator performed all pQCT scans, and one technician performed all scan analyses. Repeat scanning of girls to establish the precision of pQCT was not considered ethical by the University of Arizona Human Subjects Protection Committee. Thus we conducted a separate study with adults to determine within-subject (*n* = 29 per skeletal site) pQCT precision error (CV). After subject repositioning, CVs calculated as described by Glüer and colleagues(38) for trabecular vBMD at the 4% femur and tibia were 0.5% and 0.8%, respectively. CVs for femur and tibia PC were 0.4% and 1.1%, respectively. DXA CVs and BMD precision in our laboratory have been reported previously.(39)

### Statistical analysis

All data were analyzed using the Statistical Package for the Social Sciences for Windows, Version 18.0 (SPSS, Chicago, IL, USA). Data were checked for outliers and normality using histograms, and all variables were tested for skewness and kurtosis. Descriptive statistics were calculated for the entire sample and for girls with and without prior fracture. Differences in descriptive characteristics between groups were tested using the independent-samples *t* test or the chi-square test for proportions as appropriate. Bone parameters assessed by pQCT were compared between groups using analysis of covariance (ANCOVA), adjusting for maturity offset, body mass, leg length, ethnicity, and physical activity. Adjusted odds ratios (95% confidence intervals) were computed using binary logistic regression to evaluate the relationship between pQCT bone variables and prior fracture. Regression models were adjusted for maturity offset alone (Table 3, model^a^) and for maturity offset, body mass, leg length, ethnicity, and physical activity (Table 3, model^b^) to determine whether adjustment for additional covariates influenced the relationship between fracture and bone parameters. To examine the relationship between prior fracture and bone parameters assessed by DXA independently of bone and body size, BMC was adjusted for BA, body mass, and height using multiple regression, as described by Prentice and colleagues.(40) Adjusted BMC variables were compared between girls with and without prior fracture using ANCOVA, and adjusted odds ratios (95% confidence intervals) were computed using binary logistic regression employing the same covariates, except for body mass and leg length because the BMC variables already had been adjusted for body size. All ANCOVA and regression analyses were repeated substituting maturity offset with Tanner stage. All results were similar; thus we report analyses that included maturity offset based on its higher relation with all bone parameters.(22) A significance level of *p* < .05 (two-tailed) was used in all tests.

**Table 3:** Associations Between DXA and pQCT Parameters and Prior Fracture in Girls With (*n* = 88) and Without (*n* = 377) Prior Fracture

	Model^a^	Model^b^
		
Bone measure	Adjusted Odds Ratio (95% CI)	Adjusted Odds Ratio (95% CI)
DXA		
Total Body Less Head BMC (g)/SD decrease	1.19 (0.75–1.87)	1.15 (0.73–1.82)
Lumbar Spine (L2–L4) BMC (g)/SD decrease	1.18 (0.76–1.83)	1.18 (0.76–1.83)
Femur Neck BMC (g)/SD decrease	1.00 (0.61–1.62)	1.03 (0.63–1.68)
Total Femur BMC (g)/SD decrease	0.87 (0.52–1.46)	0.87 (0.52–1.47)
Total Tibia BMC (g)/SD decrease	0.95 (0.59–1.53)	0.94 (0.58–1.52)
pQCT Femur		
4% Trab vBMD (mg/cm^3^)/SD decrease	1.46 (1.13–1.89)*	1.43 (1.10–1.87)*
4% PC (mm)/SD decrease	0.96 (0.66–1.39)	1.03 (0.69–1.54)
20% Cort vBMD (mg/cm^3^)/SD decrease	0.90 (0.71–1.15)	0.85 (0.65–1.09)
20% EC (mm)/SD decrease	0.90 (0.66–1.24)	0.95 (0.67–1.34)
20% PC (mm)/SD decrease	0.90 (0.61–1.33)	0.96 (0.61–1.50)
20% Cort Thk (mm)/SD decrease	1.09 (0.84–1.42)	0.85 (0.65–1.09)
pQCT Tibia		
4% Trab vBMD (mg/cm^3^)/SD decrease	1.32 (1.03–1.70)*	1.33 (1.03–1.72)*
4% PC (mm)/SD decrease	0.92 (0.64–1.32)	0.98 (0.65–1.48)
66% Cort vBMD (mg/cm^3^)/SD decrease	0.80 (0.61–1.05)	0.72 (0.54–0.97)*
66% EC (mm)/SD decrease	1.07 (0.82–1.40)	1.13 (0.85–1.50)
66% PC (mm)/SD decrease	1.07 (0.77–1.49)	1.18 (0.81–1.74)
66% Cort Thk (mm)/SD decrease	0.95 (0.71–1.25)	0.94 (0.70–1.25)

Values are presented as adjusted odds ratios (95% CI). *p *<* 0.05.

BMC = bone mineral content; Trab vBMD = trabecular volumetric bone mineral density; PC = periosteal circumference; Cort vBMD = cortical volumetric bone mineral density; EC = endosteal circumference; Cort Thk = cortical thickness.

a Model = maturity offset.

b Model = maturity offset, body mass, leg length, ethnicity, and physical activity. Binary logistic regression models that included DXA adjusted BMC variables did not have body mass and leg length as covariates.

## Results

A total of 465 girls were measured (response rate 63% of those contacted). Sample ethnicity was 22% Hispanic and 78% non-Hispanic; race was 88% white, 7% Asian, 3% black or African American, 0.5% Native American or Alaska Native, 0.5% Native Hawaiian or other Pacific Islander, and 1% other. Tanner stage distributions for the total sample were 33% prepubertal (stage I, *n* = 155), 60% early pubertal (stages II–III, *n* = 280), and 7% late pubertal (stages IV–V, *n* = 30). Maturity offset values indicated that girls were, on average, 1.1 years from PHV, with a range from 3.2 years prior to PHV to 1.4 years after PHV. Based on U.S. National Center for Health Statistics/Center for Disease Control and Prevention percentiles for body mass index (BMI, kg/m^2^),(41) 3.0% of the sample was underweight (BMI < 5th percentile), 73.8% of the sample was healthy weight (BMI 5th to 85th percentile), 15.1% of the sample was overweight (BMI 85th to 95th percentile), and 8.1% of the sample was obese (BMI > 95th percentile).

Of the total sample, 88 girls (19%) had experienced a total of 104 fractures (Table 1). Cooper and colleagues(4) reported that approximately 33% of girls sustain at least one fracture before 17 years of age. Fracture prevalence in our young sample was lower (19%), although this could be expected because the probability of sustaining at least one fracture increases with age. In our sample, 76 girls sustained a single fracture, 8 girls had fractured twice, and 4 girls sustained three fractures. The forearm was the most common site afflicted, accounting for 49% of all fractures (Table 1). However, a number of fractures at other skeletal sites also were reported (Table 1). Age at peak prevalence of fracture was 10, when 16% of fractures occurred.

**Table 1:** Numbers of Fractures at Different Skeletal Sites for Participants With Prior Fracture (*n* = 88)

Site of fracture	1st fracture (*n* = 88)	2nd fracture (*n* = 12)	3rd fracture (*n* = 4)	All fractures (*n* = 104)
Clavicle	7 (8.0%)	1 (8.3%)		8 (7.7%)
Femur	1 (1.1%)			1 (1.0%)
Fingers/thumb	13 (14.8%)	1 (8.3%)		14 (13.5%)
Foot/ankle	10 (11.4%)	2 (16.7%)		12 (11.5%)
Hand	1 (1.1%)			1 (1.0%)
Humerus	4 (4.5%)			4 (3.8%)
Radius/ulna	39 (44.3%)	8 (66.7%)	4 (100%)	51 (49.0%)
Skull	2 (2.3%)			2 (1.9%)
Tibia/fibula	8 (9.1%)			8 (7.7%)
Toes	3 (3.4%)			3 (2.9%)

Values are presented as number of fractures and percent in parentheses.

Descriptive characteristics are shown in Table 2. Girls with prior fracture were significantly older and more mature, as assessed by maturity offset and Tanner stage, and had significantly greater body mass, height, and leg length. No differences were observed between girls with and without fracture in smoking habits, ethnicity, BMI, percent body fat, and physical activity score.

**Table 2:** Descriptive Characteristics

	All subjects (*n* = 465)	Subjects with fracture (*n* = 88, 19%)	Subjects without fracture (*n* = 377, 81%)	*P*
Smoking (%)	0.7%	0.0%	0.8%	.40
Ethnicity (%; Hispanic)	22%	18%	24%	.29
Age (years)	10.6 ± 1.1	11.0 ± 1.0	10.5 ± 1.1	<.001
Tanner Stage (1–5)	2.1 ± 1.0	2.5 ± 0.9	2.0 ± 1.0	<.001
Maturity Offset (years)	−1.1 ± 1.0	−0.6 ± 1.0	−1.2 ± 1.0	<.001
Body mass (kg)	39.2 ± 10.4	43.3 ± 11.0	38.3 ± 10.0	<.001
Height (cm)	144.4 ± 9.8	149.1 ± 9.5	143.3 ± 9.5	<.001
BMI (kg/m^2^)	18.6 ± 3.4	19.3 ± 3.7	18.4 ± 3.3	.03
Leg length (cm)	68.9 ± 5.7	71.5 ± 5.2	68.2 ± 5.6	<.001
Femur length (cm)	34.0 ± 3.0	35.3 ± 2.6	33.7 ± 3.0	<.001
Tibia length (cm)	33.2 ± 2.8	34.5 ± 2.7	32.9 ± 2.7	<.001
Body fat (%)	27.8 ± 8.4	29.7 ± 8.8	27.4 ± 8.3	.02
Physical activity score^a^	860.6 ± 918.2	837.2 ± 638.1	866.2 ± 973.4	.79

Values are presented as percent or mean ± SD. Differences in descriptive characteristics between the two groups were investigated using independent samples *t*-test, except for smoking and ethnicity where X^2^ was used.

a Physical activity score = ∑1–*n* (Duration × Frequency × Load).

**Fig. 1:**
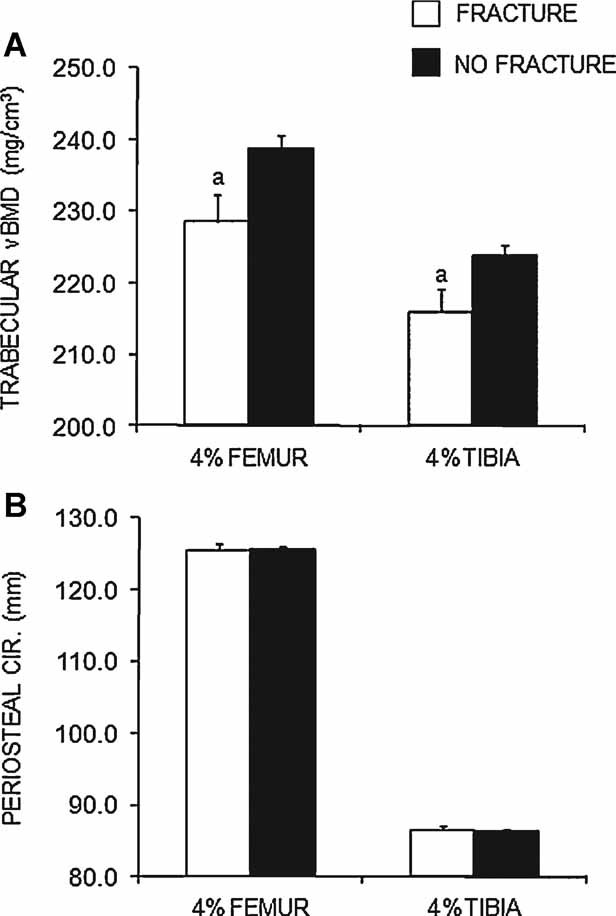
Metaphyseal trabecular vBMD (*A*) and periosteal circumference (*B*) in girls with (*n* = 88) and without prior fracture (*n* = 377). Differences in bone variables were evaluated by ANCOVA using maturity offset, body mass, leg length, ethnicity, and physical activity as covariates. Bars represent adjusted means ± SE. ^a^*p* < .05.

ANCOVA, with maturity offset, ethnicity, and physical activity as covariates, was used to investigate possible differences in bone parameters assessed by DXA between girls with and without fracture. Girls with prior fracture had 0.9% to 1.8% lower adjusted BMC of the TBLH, LS, and FN regions than girls without prior fracture, although the difference in adjusted BMC was not statistically significance at any region. ANCOVA, with maturity offset, body mass, leg length, ethnicity, and physical activity as covariates, also was used to investigate possible differences in bone parameters assessed by pQCT between girls with and without fracture. At distal metaphyseal sites, trabecular vBMD of the femur and tibia was significantly lower (femur = –4.5%, tibia = –3.1%) in girls with prior fracture (Fig. 1). At diaphyseal sites of the femur and tibia, cortical vBMD, EC, PC, and cortical thickness were not significantly different between girls with and without fracture.

To evaluate the association between prior fracture and DXA and pQCT bone outcomes, adjusted odds ratios (95% confidence interval) were computed using binary logistic regression (Table 3). Regression models adjusted for maturity offset alone (Table 3, model^a^) and all covariates (Table 3, model^b^) resulted in nonsignificant associations between BMC (adjusted for BA, body mass, and height) assessed by DXA and prior fracture at all sites. In contrast, lower trabecular vBMD at metaphyseal regions of the distal femur and tibia remained significantly associated with fracture (Table 3). After adjustment for all covariates (Table 3, model^b^), every SD decrease in trabecular vBMD at metaphyseal sites of the femur and tibia was associated with 1.4 (1.1–1.9) and 1.3 (1.0–1.7) times higher fracture prevalence, respectively. Metaphyseal PC of the distal femur and tibia was not associated with fracture (Table 3). At diaphyseal sites of the femur and tibia, EC, PC, and cortical thickness were not associated with facture, although every SD decrease in cortical vBMD of the 66% tibia but not the 20% femur was associated with lower fracture prevalence [odds ratio (OR) = 0.72, 95% confidence interval (CI) 0.54–0.97, *p* = .032]. Adjusted odds ratios (95% CI) also were computed using binary logistic regression to assess whether DXA and pQCT bone parameters were associated with prior tibial fracture (*n* = 8). Regression models adjusted for maturity offset alone and all covariates resulted in nonsignificant associations between bone parameters of the nondominant tibia and prior tibial fracture (data not shown).

## Discussion

This study investigated the relationship between prior fracture and bone parameters using pQCT and DXA in 465 pre- and peripubertal girls aged 8 to 13 years. We found that prior fracture was associated with lower trabecular vBMD (assessed by pQCT) at distal metaphyseal sites of the femur (–4.5%) and tibia (–3.1%). In contrast, BMC (assessed by DXA) adjusted for BA, body mass, and height and bone geometric parameters (assessed by pQCT) showed no differences between girls with and without prior fracture. These findings suggest that fractures during growth are not related to smaller bone size and that vBMD is more highly associated with prior fracture than BMC adjusted for bone and body size. These results add to the expanding body of evidence linking lower vBMD at metaphyseal regions of long bones with fractures during growth.(16,18)

Previous studies in children and adolescents of fracture and bone parameters using 3D imaging techniques are few, and thus far, all have focused on the forearm.(16,20,21) In a sample of 337 children and adolescents (aged 6 to 18 years; *n =* 171 girls), Rauch and colleagues,(20) using pQCT, reported that endocortical apposition was not increased sufficiently to keep cortical thickness adapted appropriately to increases in mechanical challenges from increasing bone length and body mass. The authors concluded that a lag in cortical thickness during growth may explain the high prevalence of forearm fractures during growth.(20) Unfortunately, Rauch and colleagues did not relate fracture prevalence with pQCT parameters, making it impossible to compare our results with that study.(20) In 100 healthy white girls (aged 4 to 15 years), Skaggs and colleagues(21) used computed tomography (CT) to show that forearm fractures were associated with lower radial CSA and higher body mass. Furthermore, trabecular vBMD at metaphyseal sites and cortical vBMD at diaphyseal sites were not significantly different between girls with and without forearm fracture, although girls who had a fracture had approximately 3% lower trabecular vBMD at distal metaphyseal sites of the radius.(21) It is possible the study was underpowered (*n* = 50 per group) to detect significant between-group differences in trabecular vBMD. More recently, in a sample of 396 pubertal Finnish girls (aged 10 to 13 years), Cheng and colleagues(16) reported approximately 10% lower radial vBMD in girls with prior fracture compared with girls without prior forearm fracture. Furthermore, in a population-based cohort study of young-adult men (aged ∼19 years), Darelid and colleagues(18) reported that prior fracture was associated with lower trabecular vBMD at the distal radius (−6.6%) and tibia (−4.5%). These findings agree with our findings of lower trabecular vBMD at the distal femur (−4.5%) and tibia (−3.1%), suggesting a consistent association between fracture during growth and lower trabecular vBMD at metaphyseal sites of long bones. In a recent study using high-resolution pQCT (HR-pQCT), Kirmani and colleagues(42) showed that trabecular bone parameters (bone volume fraction, trabecular number, and thickness) at distal metaphyseal regions of the radius remained stable throughout puberty in girls. Thus assessment of metaphyseal trabecular bone parameters during growth may be an important strategy for identifying girls at risk for fractures so that appropriate interventions can be delivered.

In contrast to the relationship found between lower trabecular vBMD at metaphyseal regions of the femur and tibia and higher fracture prevalence, we found that lower cortical vBMD at the diaphyseal site of the tibia was associated with lower fracture prevalence. This finding should be interpreted with caution. A similar association was not significant at the diaphyseal site of the femur. Moreover, to our knowledge, no previous studies have reported a similar finding, and we know of no plausible reason for a different association at the diaphyseal site of the tibia. Future studies of the relationship between fractures and diaphyseal cortical vBMD are needed to test whether this is a consistent finding or whether it occurred by chance.

To date, studies of fractures in youth using DXA have given conflicting results,(8–12,43–45) suggesting that fracture history is related(8–12) or not related(43–45) to bone parameters. The contradictory conclusions likely occurred because there is no protocol to adequately address the limitations of DXA in children.(36) Nevertheless, in a meta-analysis of studies using DXA and other methods [ie, pQCT, quantitative ultrasound (QUS), metacarpal morphometry], Clark and colleagues(14) concluded that there may be associations between BMD, BMC, and prior fracture, although evidence was limited. To our knowledge, our study is the first in girls to relate fracture history with adjusted BMC (assessed by DXA) and vBMD (assessed by pQCT). We found that vBMD was more highly associated with prior fracture than adjusted BMC. Recently, Darelid and colleagues(18) and Taes and colleagues(46) reported in men that both vBMD (assessed by pQCT) and aBMD (assessed by DXA) were associated with prior fracture. Our findings agree with previous studies(18,46) in that lower metaphyseal vBMD has a consistent association with prior fracture, although we found only weak associations between lower BMC and fracture. These findings suggest that application of tools such as pQCT may be clinically significant for identifying girls at risk for fracture, although prospective studies will be needed to confirm that lower vBMD precedes and predicts fractures and that decreased mobilization after fracture is not responsible for reducing vBMD.

The pQCT device used in this study cannot assess cortical vBMD accurately at metaphyseal regions of long bones because the spatial resolution of the instrument is not sufficient to analyze cortical shells less than 2 mm.(47) Thus we assessed trabecular vBMD at metaphyseal sites and cortical vBMD at diaphyseal sites of the femur and tibia. Recent studies in postmenopausal women have used HR-pQCT to assess micro- and macrostructural variables, along with trabecular and cortical vBMD at the ultradistal radius (UDR).(48,49) Melton and colleagues(48) used DXA and HR-pQCT to show that postmenopausal women with a history of fracture had 10% lower aBMD of the arms and 10% lower cortical vBMD of the UDR. Interestingly, 22% lower trabecular vBMD of the UDR was observed between women with prior forearm fracture compared with controls, which suggests a stronger relationship between fractures and reduced trabecular vBMD at the UDR. Sornay-Rendu and colleagues(49) reported similar findings using HR-pQCT in postmenopausal women. Moreover, both the studies by Melton and colleagues(48) and Sornay-Rendu and colleagues(49) showed that HR-pQCT parameters at the distal tibia were associated with fractures with similar magnitude to the parameters at the UDR. In toto, the findings from our study and other studies in youth,(16) young adults,(18,46) and postmenopausal women(48,49) support the premise that lower trabecular vBMD of the radius, femur, and tibia is associated with prior fracture, which suggests that trabecular vBMD at any given metaphyseal site is relatively representative of other skeletal regions.

This study had some limitations. The main limitation is that our data are cross-sectional, and therefore, we cannot assess whether lower trabecular vBMD is causative rather than consequential. However, lower trabecular vBMD could be causative because few fractures resulted in casting or immobilization of the femur and tibia and physical activity levels were not different between girls with and without prior fracture. Another limitation is that fractures may have been misreported. Unfortunately, we did not have access to medical charts or radiographs to confirm reports. However, any fracture misclassifications likely would have lead to an underestimated association between bone parameters and fracture. Also, bone measurements were taken at variable times after fracture; thus different intervals between fracture and bone measurements may have influenced our results. Lastly, our pQCT measurements were limited to the femur and tibia, and the pQCT device used in this study cannot accurately assess cortical vBMD at metaphyseal regions of long bones because of limitations in spatial resolution.(47) Future studies using HR-pQCT in children and adolescents will be necessary to determine whether fractures are associated with microstructural variables, along with cortical vBMD at metaphyseal regions of long bones.

Despite these limitations, the study had several significant strengths. First, the study evaluated the relationship between prior fracture and vBMD and bone geometry at metaphyseal and diaphyseal sites of the femur and tibia. The relationship between prior fracture and pQCT parameters at these sites has not been studied previously in children. Our results at these sites are consistent with those reported at the radius,(16) the most commonly reported fracture site in children, suggesting that the relationship between prior fracture and lower trabecular vBMD at metaphyseal regions of long bones is consistent throughout the appendicular skeleton. The potential poor long-term reproducibility of pQCT in assessing metaphyseal bone size and vBMD from a single-slice measurement has been a concern of many previous studies because bone size and vBMD vary greatly along metaphyseal regions of long bones.(34) Recognizing this challenge, we averaged three pQCT slices at metaphyseal regions, which improved our estimates of precision (trabecular vBMD CVs at the distal femur and tibia were 0.5% and 0.8%, respectively).

In conclusion, fractures in girls are associated with lower trabecular vBMD at distal metaphyseal sites of the femur and tibia. Furthermore, fractures are more strongly related to vBMD (assessed by pQCT) compared with adjusted BMC (assessed by DXA). These finding are consistent with previous studies using pQCT, which have reported that fracture history is associated with lower trabecular vBMD at distal metaphyseal sites of the radius during growth,(16) during young adulthood,(18,46) and after menopause.(48,49) These findings suggest that the relationship between fracture and lower trabecular vBMD at metaphyseal sites tracks throughout life and is similar among weight-bearing and non-weight-bearing long bones. Therefore, lower trabecular vBMD at metaphyseal sites of long bones may be an early marker of skeletal fragility in girls.
